# Pleural Mycobacterium Avium Complex Infection in an Immunocompetent Female with No Risk Factors

**DOI:** 10.1155/2015/760614

**Published:** 2015-02-22

**Authors:** Ravi P. Manglani, Misbahuddin Khaja, Karen Hennessey, Omonuwa Kennedy

**Affiliations:** Lincoln Medical and Mental Health Center, Department of Internal Medicine, New York, NY 10451, USA

## Abstract

Mycobacterium avium complex (MAC) infections rarely affect the pleura, accounting for 5–15% of pulmonary MAC. We report a case of MAC pleural effusion in an otherwise immunocompetent young patient. A 37-year-old healthy female with no past medical history was admitted to the hospital with two weeks of right sided pleuritic chest pain, productive cough, and fever. She was febrile, tachycardic, and tachypneic with signs of right sided pleural effusion which were confirmed by chest X-ray and chest CT. Thoracentesis revealed lymphocytic predominant exudative fluid. The patient underwent pleural biopsy, bronchoscopy with bronchoalveolar lavage, and video assisted thoracoscopic surgery (VATS), all of which failed to identify the causative organism. Six weeks later, MAC was identified in the pleural fluid and pleural biopsy by DNA hybridization and culture. The patient was started on clarithromycin, ethambutol, and rifampin. After six months of treatment, she was asymptomatic with complete radiological resolution of the effusion. The presence of lymphocytic effusion should raise the suspicion for both tuberculous and nontuberculous mycobacterial disease. Pleural biopsy must be considered to make the diagnosis. Clinicians must maintain a high index of suspicion of MAC infection in an otherwise immunocompetent patient presenting with a unilateral lymphocytic exudative effusion.

## 1. Introduction

Nontuberculous mycobacteria were first described soon after Robert Koch's discovery of tuberculous Bacilli in the late 19th century. They were not identified as clinically important pathogens until the advent of HIV and patients with severely compromised immune systems [1]. The clinical presentations of pulmonary mycobacterium avium complex (MAC) infections in immunocompetent hosts may be classified as those with (a) preexisting lung disease, (b) no preexisting lung disease, and (c) atypical and rare presentations. Pleuritis and pleural effusions are included under rare and atypical presentations [2, 3], accounting for 5–15% of pulmonary MAC [4, 5].

## 2. Case Report

**Figure 1:**
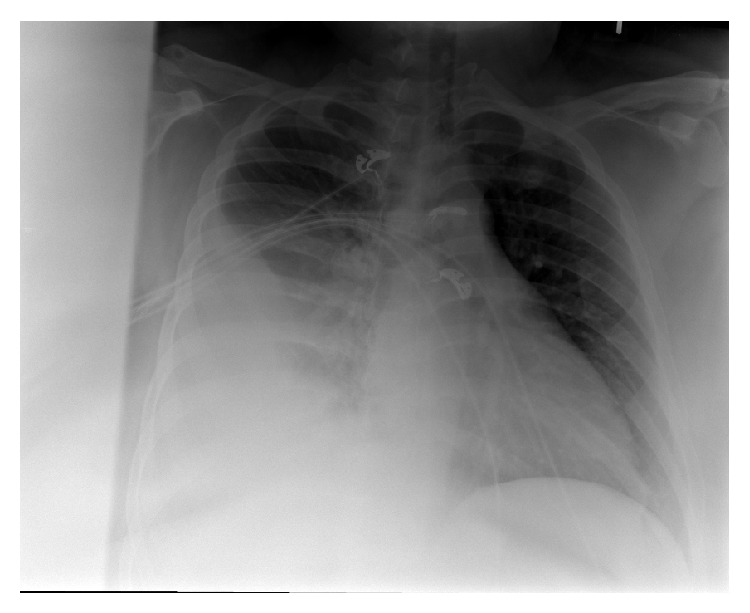
Chest X ray on presentation.

**Figure 2:**
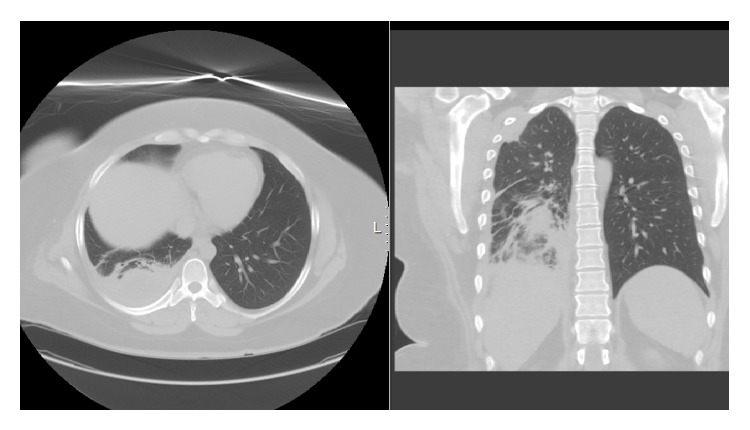
Chest CT on presentation.

The patient is a 37-year-old female, admitted to the hospital with complaints of two weeks duration of right sided pleuritic chest pain, productive cough, and fever, after a trip to Algeria. On examination, the patient is an obese female, febrile, tachycardic, tachypneic, and was found to have signs of right sided pleural effusion (Figure 1), confirmed by chest X-ray. Chest CT revealed a large right sided pleural effusion with compressive atelectasis, right upper lobe pneumonia, right middle lobe nodule, and a chain of pretracheal lymph nodes that showed central necrosis (Figure 2).

Thoracentesis revealed lymphocytic predominant exudative fluid. The fluid was cloudy yellow, with WBC count of 140 with 22% segmented neutrophils, 54% lymphocytes, and 24% monocytes. Adenosine deaminase was 46.4 (elevated), fluid lactate dehydrogenase was 190, pH was 7, fluid glucose was 34, fluid albumin was 2.7, and fluid chloride was 115. Given this fact, her recent travel history, symptom profile, and radiological features, the patient was placed on respiratory isolation for suspicion of tuberculous disease. Three sputum samples were obtained for testing for acid fast Bacilli, all of which were negative.

The patient subsequently underwent pleural biopsy, bronchoscopy with bronchoalveolar lavage, and video assisted thoracoscopic surgery, all of which failed to provide a causative organism. Six weeks later, mycobacterium avium complex was isolated from pleural fluid and pleural biopsy specimens, by DNA hybridization.

**Figure 3:**
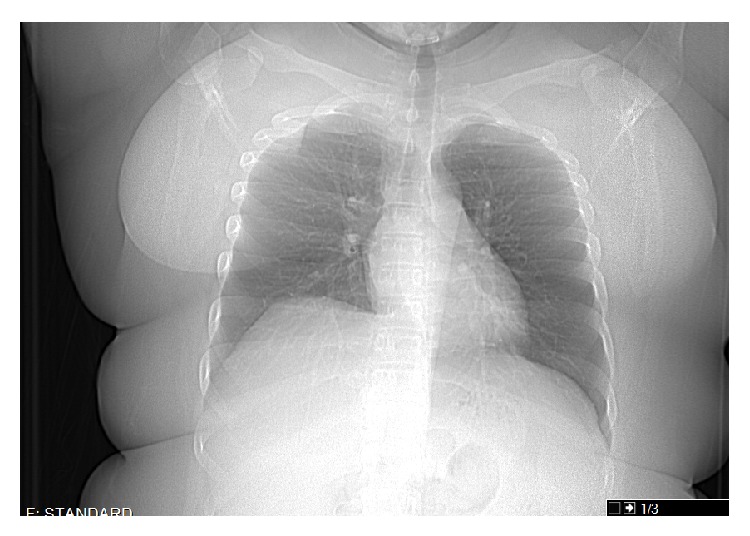
Chest X ray after treatment.

**Figure 4:**
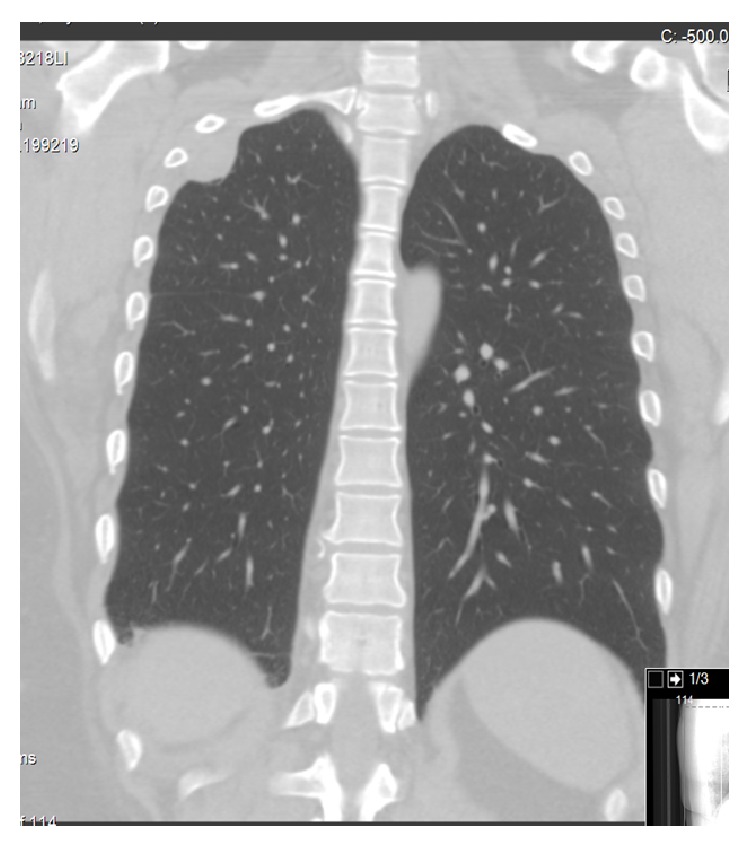
Chest CT after treatment.

The patient was started on clarithromycin, ethambutol, and rifampin and continues to take therapy to complete twelve months of treatment. She has been asymptomatic for six months. Subsequent follow up chest X-ray and chest CT shows complete resolution of the pleural effusion (Figures 3 and 4).

## 3. Discussion

Nontuberculous mycobacteria are naturally occurring organisms present in water, soil, and biofilms all over our environment. They do not ordinarily cause disease in the immunocompetent population, especially those without risk factors of chronic lung diseases. The predisposing factors include chronic obstructive pulmonary disease, lung fibrosis, lung cancer, cystic fibrosis occupational lung diseases, and others. Transmission of the pathogen is not well described, although person-to-person transmission is unlikely, especially in immunocompetent hosts [6, 7].

Pleural fluid growing MAC has been described in unrelated diseases the past, such as congestive heart failure and malignancy. These results are not regarded as clinically significant. However, if MAC is isolated from an enclosed space, it is generally regarded as the causative organism for the pathology observed [2]. Our patient did not present with features typical of the well described “Lady Windermere Syndrome.” This syndrome describes a constellation of reticulonodular infiltrates, cylindrical bronchiectasis involving the right middle lobe or lingular lobe bronchiectasis in a middle aged woman due to MAC infection [8].

The patient was not in the habit of taking long baths in hot tubs. The presentation of “Hot tub lung” is more insidious and dominated by dyspnea, cough, low grade fever, and diffuse infiltrates on chest X-ray. She presented with pleuritis and pleural effusion in the absence of other pulmonary risk factors. The patient was in no way immunocompromised and so did not present with disseminated disease.

Pleural effusion and pleuritis caused by MAC in an immunocompetent host are rare and can prove to be a diagnostic challenge for physicians, especially in the absence of disseminated disease. The presence of Lymphocytic effusion must give clinicians a clue into the diagnosis. Pleural biopsies must be considered to make the diagnosis, especially if prior studies have been negative.

Treatment consists of a macrolide like clarithromycin, in addition to ethambutol and rifampin, with close follow-up for a period of twelve months. During and after treatment, proof of cure is obtained with sputum cultures and acid fast Bacilli detection on sputum.

## 4. Conclusion

MAC pleuritis and associated pleural effusions are rare [2–5] and can prove to be a diagnostic challenge, requiring a high index of suspicion. Pursuit of pleural biopsies to identify the causative organisms is warranted in cases where initial lesser invasive tests have proved unyielding.
